# Review of respiratory syndromes in poultry: pathogens, prevention, and control measures

**DOI:** 10.1186/s13567-025-01506-y

**Published:** 2025-05-17

**Authors:** Huixin Liu, Sijia Pan, Chenchen Wang, Wenwen Yang, Xiaofang Wei, Yang He, Ting Xu, Kaichuang Shi, Hongbin Si

**Affiliations:** 1 College of Animal Science and Technology, Guangxi Key Laboratory of Animal Breeding, Disease Control and Prevention, Guangxi grass station, Guangxi University, Nanning, 530004 China; 2 Guangxi Center for Animal Disease Control and Prevention, Nanning, 530001 China

**Keywords:** Respiratory syndromes, disease triggers, detection technology, vaccine development, prevention and control measures

## Abstract

Respiratory syndromes (RS) include a variety of diseases that lead to respiratory dysfunction, resulting in significant economic losses for the poultry industry. Infectious agents and unfavourable environmental factors cause these respiratory diseases, and rapid transmission, high morbidity rates, and frequent mixed infections characterise them. The challenge in preventing and treating these diseases arises from the complexity of their triggers and the potential for secondary infections. Current vaccines often do not provide effective prevention, and the overuse of certain medications can lead to increased bacterial resistance, complicating prevention and control efforts. This review article examines the common sources of respiratory infections in poultry flocks, including infectious bronchitis virus, avian influenza virus, Newcastle disease virus, infectious laryngotracheitis virus, avian metapneumovirus, pathogenic *Escherichia coli*, *Haemophilus paragallinarum*, *Mycoplasma gallisepticum*, and *Chlamydia*. It also considers non-infectious factors such as adverse environmental conditions and management errors. The article provides an updated, comprehensive overview of widespread and economically significant poultry respiratory pathogens. It briefly discusses detection technology and vaccine development based on the transmission characteristics of RS. Furthermore, it explores prevention and control measures such as combination drug strategies and antibiotic alternatives to enhance understanding and implementation of effective disease prevention and control measures.

## Introduction

Unusually high rates of respiratory disease and mortality in the poultry industry are often reported, particularly in certain countries where most outbreaks and deaths are associated with a complex respiratory syndrome [1]. Various clinical symptoms characterise this syndrome, and it has an unclear aetiology. The respiratory mucosal immune system in poultry has a comprehensive network throughout the body, serving as a critical defence barrier against infections. Respiratory syndromes (RS) are a significant concern in poultry, arising from complex interactions between pathogens and environmental factors. Breathing difficulties due to respiratory diseases are a leading cause of mortality in poultry [2]. Additionally, mixed infections with different pathogens can have synergistic effects, compromising the mucosal immune defences of the respiratory tract and potentially exacerbating respiratory disease [3, 4].

**Figure 1:**
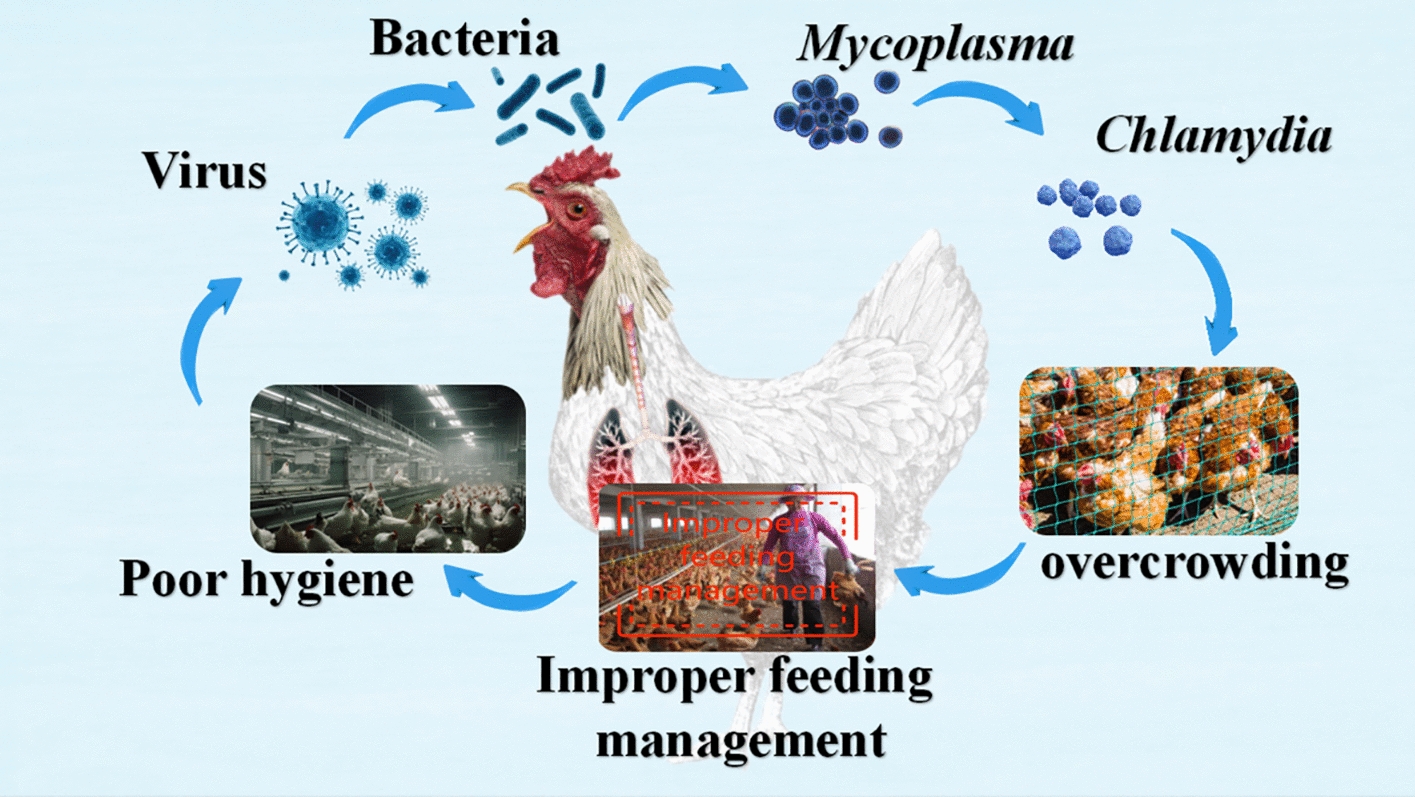
**The most common cause of respiratory infections in poultry.**

In poultry, RS can be caused by both infections and non-infectious factors. Infectious factors include viruses, bacteria, *Mycoplasma*, and *Chlamydia*, while non-infectious factors often stem from poor hygiene and improper feeding management (Figure 1). Viruses, bacteria, and *Mycoplasma* are the main agents responsible for respiratory disease syndrome in chickens. Key pathogens include the infectious bronchitis virus (IBV), Avian Influenza Virus (AIV), Newcastle disease virus (NDV), and infectious laryngotracheitis virus (ILTV) [5, 6].

Poultry respiratory syndrome tends to spread rapidly and has a high incidence rate. Common symptoms in chicks include coughing, sneezing, difficulty breathing, rales, runny nose, eye swelling, and tearing of the eyes. In adult chickens, the disease can lead to reduced egg production and quality [7, 8]. Anatomical examinations may reveal pathological changes such as laryngeal and tracheal bleeding, increased respiratory mucus, blockages in the breast, turbidity of the air sac walls, and pulmonary necrosis [9, 10].

The damage caused to the respiratory system by RS leads to decreased production performance and higher mortality rates in chickens. RS is difficult to eradicate and often recurs, resulting in significant economic losses for farmers. Maintaining respiratory health is essential for optimal performance in commercial poultry, highlighting the need for adequate surveillance programs and global cooperation [11, 12]. This study aims to outline the current prevalence of RS and provide a comprehensive overview of the causes, prevention, and control strategies for common avian respiratory diseases in poultry flocks. This information will improve understanding and enhance prevention and control measures for this disease.

## Related reports of RS

Respiratory tract infections pose a significant threat to the poultry industry due to the involvement of multiple pathogens that cause respiratory disease syndromes in birds [13]. Co-infections are common and often lead to more severe clinical outcomes than single infections. The bidirectional synergistic interactions between these concurrent respiratory pathogens contribute to severe clinical manifestations and high mortality rates.

For example, H9N2 AIV infection can upregulate the expression of pro-inflammatory cytokines, such as interferons (IFN-γ and IFNα) and interleukins (IL-17A and IL-22), while simultaneously decreasing the production of essential gel-forming mucins, endogenous cloverleaf expression factor family peptides, and tight junction proteins (ZO-1, claudin 3, and occludin) [14]. This imbalance results in significant intestinal damage and increased proliferation of Proteobacteria, particularly *Escherichia coli*.

Concurrent infection with respiratory viruses and Avian Pathogenic *Escherichia coli* (APEC) has a more significant impact on the respiratory tracts of chickens compared to a single APEC infection, suggesting that immunopathogenesis plays a role in the persistence of lesions [15]. Furthermore, co-infection with IBV and APEC can lead to chronic complications and significant economic losses in the global poultry industry.

Transcriptome studies indicate that the co-infected group demonstrates heightened immune responses and increased macrophage infiltration. Specifically, this group shows upregulation of key pathways, including apoptotic, cytokine-mediated signalling, and PAMPs recognition clusters [16].

As a secondary pathogen, avian metapneumovirus (aMPV) disrupts bacterial attachment. Sequential infection with avian metapneumovirus followed by *Mycoplasma gallisepticum* leads to prolonged viral replication, an enhanced innate immune response [17]. Increased arachidonic acid metabolism and the induction of LTC4 in serum may serve as potential biomarkers for respiratory disease detection in poultry.

Concurrent respiratory diseases, such as *Mycoplasma gallisepticum*, *Mycoplasma synoviae*, and infectious rhinitis, combined with immunosuppressive conditions (such as mould poisoning, chicken anaemia virus, reticuloendotheliosis virus, and Marek’s disease virus-induced immunity suppression) can exacerbate the impact of ILT in poultry production [18].

A study conducted in 2023 screened 146 poultry birds suspected of chronic respiratory disease in the Indian states of Haryana and Rajasthan for avian *Mycoplasma*, Newcastle disease virus, infectious bronchitis virus, and other avian pathogens. The findings revealed that approximately half of the chickens were affected by avian mycoplasmosis and were co-infected with bacteria and/or viruses [19].

Another analysis of respiratory virus infections in 359 broiler flocks in Egypt, conducted from January to October 2022, identified common viral co-infections such as H9 + b, nd + b, and nd + h9 [20]. Furthermore, between 2017 and 2018, poultry production in Egypt faced challenges due to the co-circulation of various respiratory viruses, including the highly pathogenic AIV H5N1 (2.2.1.2) and low pathogenic H9N2 (GC1B) strains [21].

In 2022, Ethiopia reported moderate to high seroprevalence rates of NDV, aMPV, ILTV, IBV, and gallinoxin due to exposure to *Mycoplasma gallisepticum* (MG). Similarly, a surveillance study in Turkey during the same year found mixed infections of avian coronavirus, infectious bronchitis virus, and avian metapneumovirus in poultry that exhibited respiratory symptoms [21].

Field-collected clinical oropharyngeal swab samples from Kenyan live poultry markets in 2022 showed that all NDV-positive field samples were co-infected with one or more other respiratory pathogens [22]. A study conducted in 2020 found that at least one bacterial or viral pathogen was present in 80.3% of flocks in the Mekong Delta region of Vietnam, with 47.5% of those flocks harbouring more than one pathogen, including a high prevalence of *Faecalibacterium paragallinarum* [23].

Samples collected between 2018 and 2019 in the West Azerbaijan Province of Iran showed a prevalence of infectious vesicle disease and complications from multiple respiratory infections involving NDV and avian tuberculosis virus [24]. A 2017 study, which examined tracheal samples from poultry in France and Morocco for acute respiratory syndrome, identified widespread co-infections in most populations [25], with real-time reverse transcriptase PCR (rt-PCR) confirming the presence of at least two respiratory viruses in over 80% of groups [26].

Between 2017 and 2019, seven outbreaks of respiratory illnesses affecting both commercial and backyard poultry flocks in Slovenia were linked to *Mycoplasma synoviae*, IBV, MG, infectious laryngotracheitis, and co-infections involving fowl pox and other pathogens [27]. Additionally, investigations into respiratory diseases in chickens in India revealed a common pattern of co-infection involving *Mycoplasma*, *E. coli*, and viruses, indicating a prevalence of compound infections in actual disease cases [19].

aMPV infection in unvaccinated broiler chickens in Brazilian poultry farms has been associated with airsacculitis and increased carcass losses. Co-infection with pathogenic bacteria, particularly *Escherichia coli*, can exacerbate these effects [28]. Lastly, studies in Ethiopia showed moderate to high exposure levels to NDV, aMPV, ILTV, IBV, and MG, indicating significant seroprevalence and highlighting the widespread exposure to multiple respiratory pathogens [21].

## Analysis of infectious factors

### Infectious bronchitis virus

IBV, a member of the order *Coronavirales*, is a single-stranded, positive-sense RNA virus with a genome length of approximately 27.6 kb. It encodes both structural proteins (S, E, M, N) and non-structural proteins (1a, 1b) [29]. Based on serological characteristics and genetic differences, IBV can be divided into various serotypes and subtypes. The primary distinction among serotypes lies in the sequence of the S protein gene. Various subtypes, such as Mass-type, Gray-type, and APN-type, have been identified, showing regional and temporal variations [30, 31].

Recent studies have indicated that differences in IBV S1 genotypes—especially in N-terminal residues and hypervariable regions—may impact the pathogenesis of IBV in chickens [32]. Common serotypes include Massachusetts, Connecticut, Arkansas, 793/B, D274, and H120, with Massachusetts and 793/B being the most prevalent [33].

The QX strain of IBV, which is dominant in Asia, Europe, Africa, and the Middle East, is known for its nephropathogenic effects and shows significant sequence variations in the major antigen-coding gene compared to other serotypes [34, 35].

Coronaviruses have developed various mechanisms to manipulate the cellular environment to ensure their survival and efficient replication [36]. The S protein is IBV’s main surface antigen and is crucial for binding to host cell receptors. The E protein is a transmembrane protein found within the inner layer of viral particles, assisting in envelope formation and the packaging of nucleic acids. The M protein is essential for viral particle formation, cell infection, and replication. The N protein, a key structural protein of IBV, protects and stabilises the viral genome, playing a significant role in replication and transcription.

Recent research has shown that the N protein of IBV targets LGP2, disrupting the chicken IFN-I signalling pathway, which allows IBV to evade the innate immune response in birds [37]. Furthermore, IBV Nsp4 can induce membrane rearrangements, forming replication organelles and facilitating viral RNA synthesis. IBV Nsp14 modulates the innate immune response by suppressing the antiviral activity of chIFN-γ, thereby inhibiting JAK-STAT signalling and affecting downstream gene expression [38]. Additionally, IBV nsp15 inhibits the formation of cytoplasmic stress granules, while IBV Nsp16 can impact the ability of host BMDCs to recognise and present antigens [39].

A study on the Massachusetts strain (M41 strain) revealed that the overlapping receptor-binding domain (RBD) within the hypervariable region (HVR) of the S protein is critical for the attachment of viral proteins. The recombinant ArkdpcS1 protein is located within this RBD [40]. Modifications to this region can enhance its binding affinity to the trachea. For QX-type IBV strains, the antigenic relatedness value may also be influenced by the HVR. Notably, variations in amino acids between S (SER) residues and other amino acid residues were observed in seven QX-type field isolates collected in China [34]. Furthermore, the binding properties of the S protein are believed to correlate with differences in pathogenicity. Minor alterations in the S1 gene can lead to antigenic variation, potentially resulting in differing vaccine responses compared to other strains.

Due to the complex epidemiology and high variability of IBV, new variants continue to emerge through genetic recombination and mutation, often lacking cross-protection between different serotypes. This suggests that vaccination against specific IBV serotypes may not protect against other variants [41, 42].

Several factors influence IBV’s pathogenicity, including its replication efficacy, tissue tropism, and ability to handle the host immune system. IBV can cause acute and highly contagious respiratory disease in chickens. Infected chickens commonly show symptoms such as tracheal rales, coughing, and sneezing [43]. The damage to the respiratory mucosa caused by IBV increases the likelihood of secondary viral and bacterial infections.

Certain strains of IBV have been found to infect blood mononuclear cells, allowing the virus to spread to internal organs such as the kidneys, lungs, fallopian tubes, and intestines. This can lead to secondary bacterial infections and exacerbate respiratory symptoms [44, 45]. While IBV can affect chickens of all ages, it is particularly severe in chicks under five weeks old. Infected chicks are more likely to exhibit noticeable respiratory symptoms than adult chickens and may experience permanent damage to their fallopian tubes. Additionally, the disease can adversely affect egg production and quality in laying hens.

**Figure 2:**
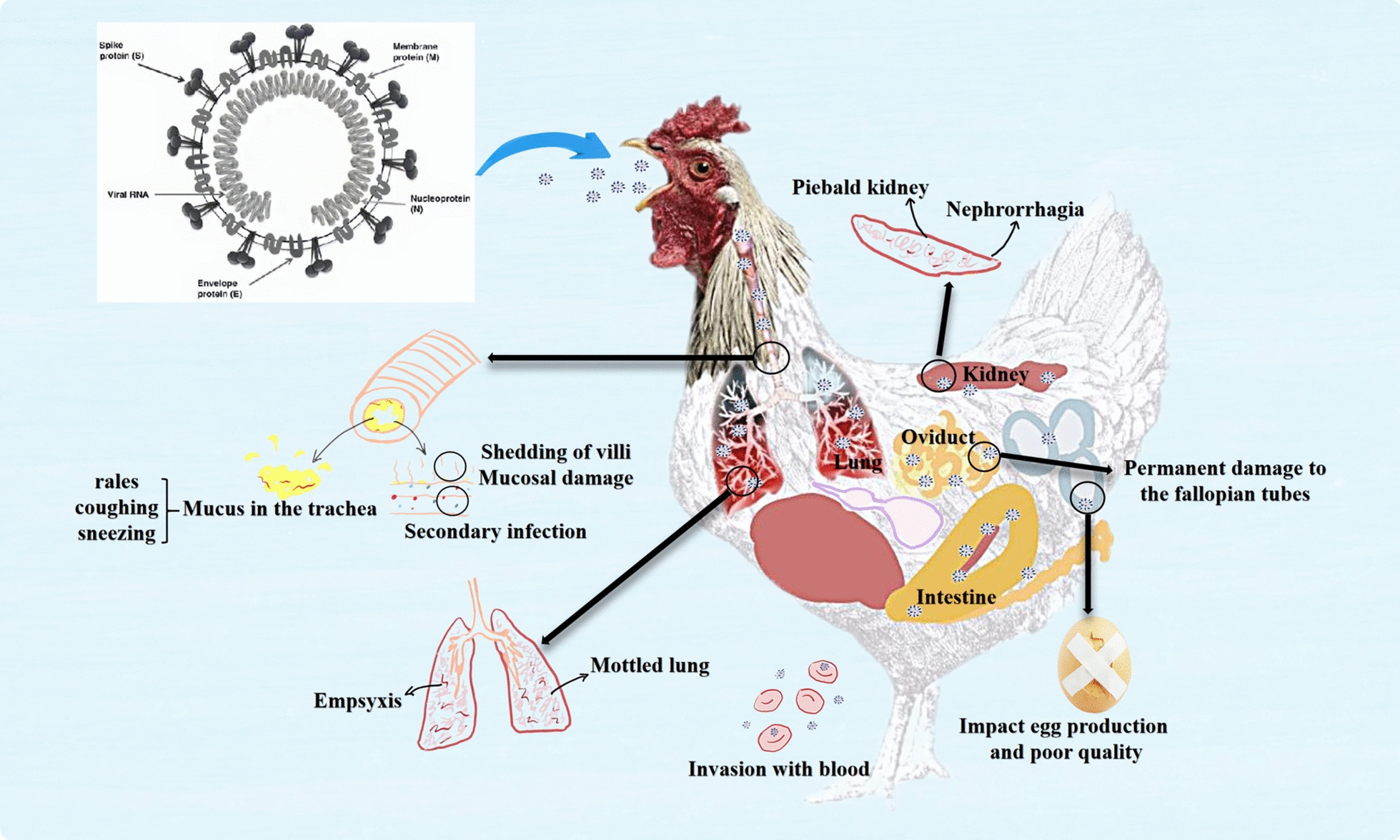
**The primary respiratory symptoms caused by IBV.**

Figure 2 illustrates the viral structure of IBV and the clinical signs and pathological lesions associated with IB syndrome. The QX type of IBV is known to utilise KUL01 + monocytes as key carrier cells, which can lead to severe kidney disease [45]. Furthermore, the QX-type of IBV elicits a more robust innate immune response and induces more significant apoptosis in CEK cells compared to the M41 strain. This difference significantly contributes to the pathological outcomes observed [35]. As a result, the QX-type of IBV demonstrates a greater ability to antagonise the host immune system response, leading to tissue lesions and potentially resulting in death.

### Avian influenza virus

AIV is a negative-stranded single-stranded RNA virus that belongs to the RNA influenza virus genus within the *Orthomyxoviridae* family. Its genome size ranges from 13 to 15 kb and consists of eight gene segments that encode 11 distinct proteins [46].

Typically, virus particles are spherical or oval, with a diameter of approximately 80 to 120 nm. AIVs exhibit two characteristic glycoprotein protrusions on their surface, known as hemagglutinin (HA) and neuraminidase (NA). HA is responsible for binding viruses to host cell receptors and helps facilitate viral-cell membrane fusion, while NA assists in the release of virus from infected cells [4].

The replication and spread of AIV can damage respiratory epithelial cells and trigger inflammatory reactions, leading to respiratory symptoms such as coughing, wheezing, and dyspnoea. The variability in the virus’s pathogenicity is attributed to the HA protein [47].

The classification of virus subtypes is based on the antigenic properties of these surface glycoproteins. To date, 18 HA subtypes (H1 to H18) and 11 NA subtypes (N1 to N11) have been identified. HA subtypes H1 to H16 and NA subtypes N1 to N9 are primarily found in birds, while H17, H18, N10, and N11 are mainly observed in bats. There is a significant diversity of variants and sublineages within each subtype, and cross-protection among them is limited [48].

The challenge in preventing and controlling AIV arises from its significant genetic variability, mainly driven by antigenic drift and antigenic rearrangement. Antigenic drift refers to point mutations occurring in viral HA and NA glycoproteins during replication, resulting in minor changes to the virus’s antigenicity. In contrast, antigenic rearrangement happens when viruses of different subtypes recombine gene segments within the same host cell, creating new virus subtypes.

These genetic variations increase the adaptability and transmissibility of AIV, posing challenges for vaccine development and antiviral drug design. Recent research has shown that AIV can undermine innate immunity through SSU72-induced transcriptional reading. Additionally, the DK1-like PB2 gene can evade the host factor SphK1’s inhibition, facilitating efficient infection [49].

Amino acid site analysis has identified A168N and D201G substitutions near the viral receptor binding site as critical determinants that alter the antigenicity of H9N2 AIV and enable immune evasion [50]. Furthermore, mutations at positions 127, 183, and 212 may also affect the antigenicity, replication, and pathogenicity of H9N2 AIV [51].

AIV is a highly contagious disease that spreads worldwide, impacting various bird and mammal populations, especially in countries with intensive poultry farming. AIV is classified into two categories based on pathogenicity: highly pathogenic avian influenza viruses (HPAIV) and low pathogenic avian influenza viruses (LPAIV) [52]. HPAIV, commonly found in the H5 and H7 subtypes, can breach respiratory and intestinal barriers, leading to high mortality rates in poultry. The H5N1 (avian flu) strain is especially devastating, causing significant economic losses with a mortality rate that can reach 100% [52]. This strain may result in various lesions in internal organs; for instance, pneumonia and pneumo-oedema are typically observed in the lungs, which can also exhibit congestion and haemorrhagic spots [53].

In contrast, LPAIV is predominantly found in wild birds and usually causes mild symptoms. However, it poses a risk of generating new reassortant viruses. Recent cases have demonstrated the evolution of new H5N1 strains through reassortment in European wild birds, presenting challenges for existing vaccines [54]. The disease affects commercial poultry and wild bird populations and has potential zoonotic implications.

**Figure 3:**
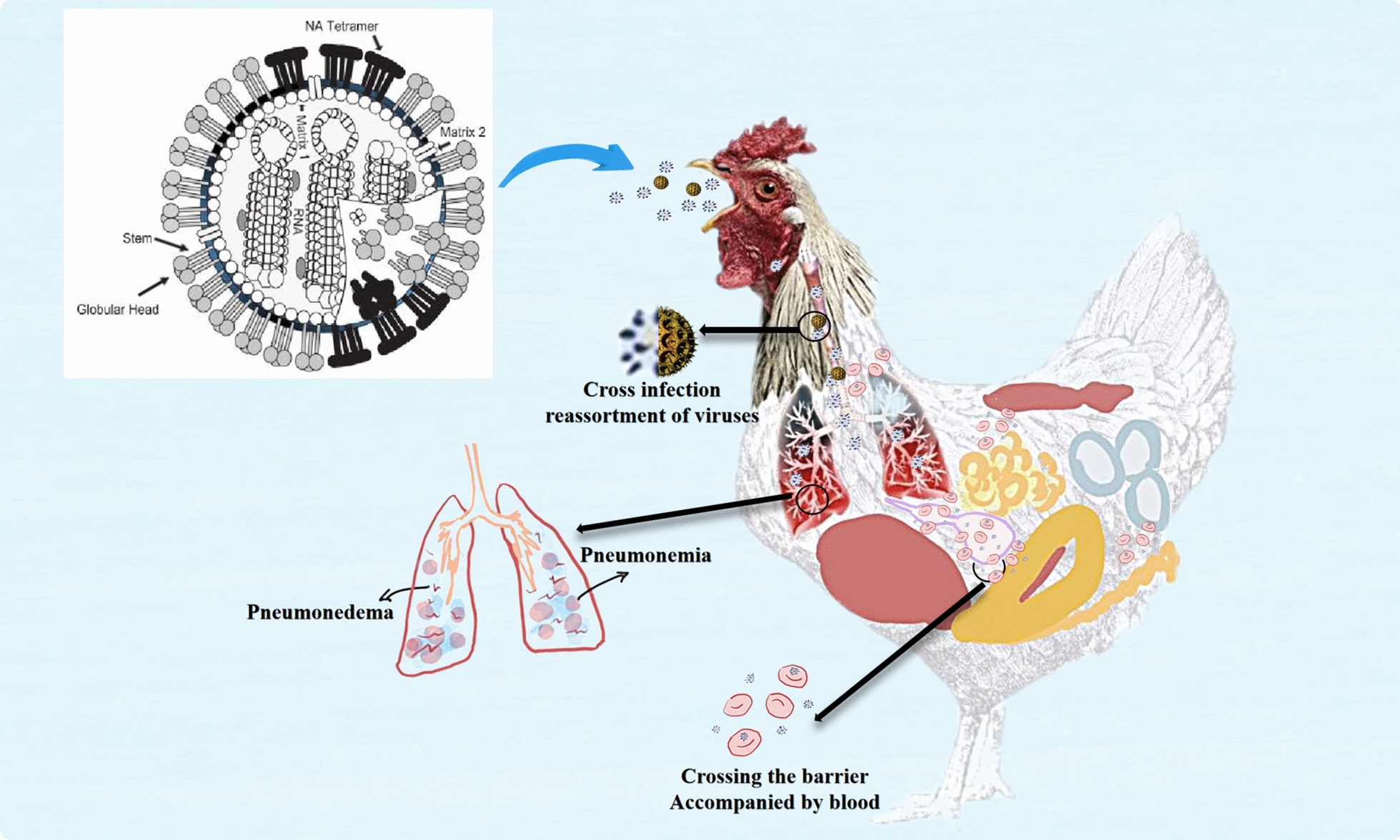
**The primary respiratory symptoms caused by AIV.**

Co-infection with different strains of HPAIV can worsen respiratory issues in poultry, while other pathogens in co-infections may enhance HPAIV replication and pathogenicity. Figure 3 illustrates the viral structure of AIV and the clinical signs and pathological changes caused by AIV in chickens. Multiple clades of AIVs can undergo genetic mutations and recombination throughout their evolution, altering their biological properties and pathogenicity. For example, the H9N2 AIV can cause more severe disease under certain conditions, even though it typically induces only mild respiratory symptoms in chickens [55]. However, H9N2 AIV often co-infects with other avian pathogens, resulting in severe clinical symptoms and high mortality rates.

Additionally, H9N2 AIV can provide internal genes for other viral subtypes, leading to viruses capable of replicating in animals or exhibiting high pathogenicity [56]. Another example is H4 AIV, which can co-circulate and recombine with other AIV subtypes, producing new viruses with increased pathogenicity, extensive host diversity, and adaptive mutations [57]. The adaptive changes in various pathogenic viruses across multiple clades may alter the virus’s affinity for host cells and enhance its pathogenicity due to genetic mutations. This can facilitate the infection of respiratory epithelial cells in poultry, resulting in severe respiratory damage, heightened inflammatory responses, and increased mortality rates.

Conversely, the virus’s evolution may enable it to evade detection and attack by the immune system, allowing it to persist and spread within the host, thereby exacerbating respiratory symptoms. Moreover, the increased host diversity during viral evolution may enable the virus to infect new host species, facilitating its spread and prevalence across different species and complicating prevention and control.

### Newcastle disease virus

NDV is part of the family *Paramyxoviridae* and the subfamily *Paramyxovirinae* [58]. The virus particles can have various shapes, including mantis-shaped or round, with diameters ranging from approximately 100 to 500 nm. These particles are enveloped, and protein-like fibres are visible on the surface of the envelope.

Viral nucleic acid consists of a single-stranded negative-sense RNA that is about 15 kb in length. The complete sequence consists of six genomes: NP (nucleoprotein), P (phosphoprotein), M (matrix protein), F (fusion protein), HN (hemagglutinin-neuraminidase protein), and L (large protein) [59, 60]. These six main structural proteins can be grouped into two categories. NP, P, and L are internal proteins involved in the transcription and replication of viral RNA, generating active mRNA. In contrast, HN, F, and M are external proteins. M is non-glycosylated and provides structural support for the inner surface of the envelope. The F protein, located on the outer surface along with the HN protein, is glycosylated and serves as a crucial protective antigen that can elicit neutralising antibodies.

NDV also produces nonstructural proteins, W and V, through a specific RNA editing mechanism involving nucleotide translocation of the P gene [61]. Despite genetic variations, all NDV isolates are serologically classified into a single serotype known as avian paramyxovirus type 1 [62]. Furthermore, based on genomic data, the fusion (F) and RNA-dependent RNA polymerase (L) genes can be classified into Class I and Class II. Class I primarily consists of attenuated types, while Class II encompasses a broader range of genotypes, with type VII being the most prevalent [63].

The long-term evolution of NDV has resulted in significant changes to its viral pathogenesis, primarily by leveraging host machinery for its replication cycle [64]. Recent studies indicate that NDV can manipulate nucleotide metabolism through the mitochondrial enzyme MTHFD2 to facilitate viral replication [65]. Additionally, it can activate multiple signalling pathways via Src and facilitate virus entry into host macrophages through pH-dependent, dynein- and Caveola-mediated endocytic pathways of Rab5 [66].

Researchers have identified various host proteins and molecules that interact with NDV proteins. For example, matrix proteins can suppress inflammatory responses by modulating the IRAK4/TRAF6/TAK1//NF-κB signalling pathway. NP proteins stimulate the PI3K/Akt/mTOR and p38 MAPK/Mnk1 pathways to promote viral mRNA translation, while the V protein disrupts mitochondrial antiviral signalling proteins and inhibits type I interferon production in the host [67]. These regulatory mechanisms play a vital role in controlling NDV infection and its pathogenicity.

The virulence of an NDV strain is crucial in determining its pathogenicity in poultry. This includes its preference for specific tissues or organs, its ability to evade the host’s immune system, and how efficiently it replicates within the host [68]. The clinical presentation of NDV infection can range from acute to subacute or chronic, with later onset symptoms affecting the respiratory, digestive, and nervous systems [69].

Less virulent strains of NDV often lead to subclinical infections, characterised by mild respiratory or intestinal issues. Moderately virulent strains can cause respiratory diseases with moderate mortality, presenting symptoms such as lacrimation, sneezing, coughing, and breathing difficulties [70]. Highly virulent strains of NDV can be categorised into two types: visceral pathogenic and neuropathogenic [71].

The visceral pathogenic type results in ulcerative bleeding, depletion of lymphoid tissue, and necrotic lesions in organs like the spleen, liver, and gut-associated lymphoid tissue. In contrast, the neuropathogenic type exhibits neurological symptoms such as severe difficulty breathing, behavioural changes, abnormal limb reflexes, head twisting, and paralysis [72].

**Figure 4:**
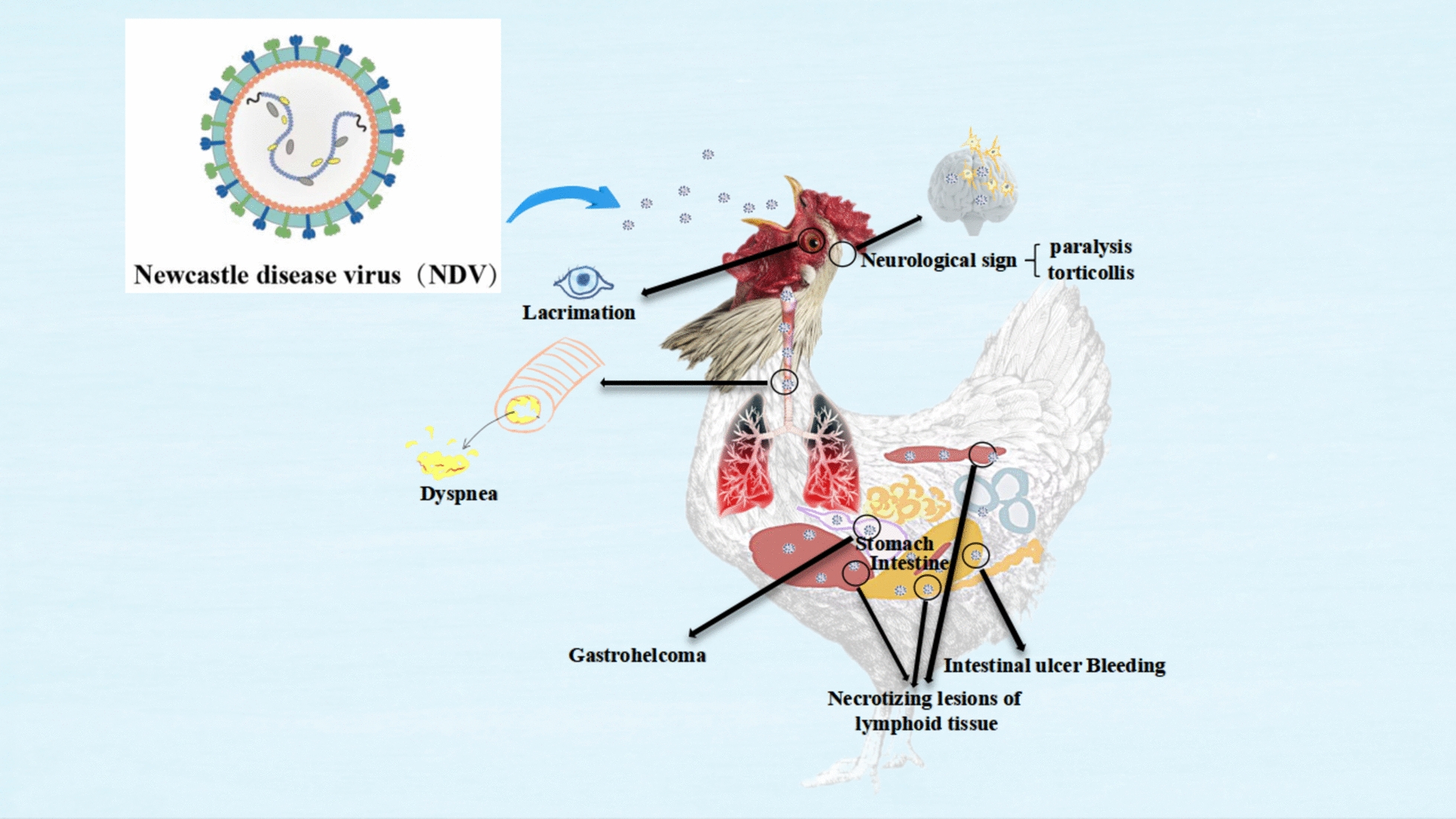
**The primary respiratory symptoms caused by NDV.**

Despite vaccination efforts, NDV continues to pose significant mortality risks to poultry flocks. Figure 4 illustrates the viral structure of NDV and the clinical signs and pathological changes caused by the virus in chickens.

### Infectious laryngotracheitis virus

ILTV, also known as Gallid herpesvirus type 1, is classified within the ILTV genus of the *Alphaherpesviridae* subfamily and the *Herpesviridae* family [73]. This double-stranded DNA virus has a genome size of approximately 152 000 base pairs. The genome includes one long (UL), one short, and two highly conserved inverted repeat (IR) sequences [74].

When examined under an electron microscope, ILTV virions reveal a structure consisting of a DNA core surrounded by an outer envelope glycoprotein, all enclosed in a membrane-coated icosahedral capsid. The viral capsid has a diameter of around 100 nm, while the complete virus particle size ranges from 200 to 350 nm.

The envelope contains various glycoproteins, including gB, gC, gD, gE, gG, gH, gI, gJ, gK, gL, and gM, all encoded by the highly conserved orf gene. Notably, glycoproteins gD, gG, gI, and gJ play essential roles in ILTV replication and in eliciting both humoral and cell-mediated immune responses in the host [75]. Previous research has shown that gD binds to host cell receptors, gG modulates the chicken immune responses, gI facilitates the spread of the virus between cells by forming heterodimers with gE, and gJ is involved in the release of the virus [76, 77].

During the first week of infection, ILTV primarily replicates in the conjunctiva and tracheal mucosa, leading to inflammation, serous or mucoid discharge, and respiratory distress [78]. The alteration of virulence by gG affects the inflammatory signalling cascades associated with the infection [79]. Recent research has shown that P53 plays a role in sustaining ILTV replication by directly regulating nucleotide metabolism and ATP synthesis through its target genes [80]. Additionally, an interaction between p53 and Fos has been identified, demonstrating a synergistic effect in regulating ILTV [81].

Genome-wide gene expression analysis has revealed that the proto-oncogene tyrosine-protein kinase Src is a crucial virulence determinant of ILTV in chicken cells, controlling the virus’s cell-to-cell spread in a manner that depends on cellular fatty acid metabolism [82]. Chip-QPCR has confirmed that Chp53 regulates the conserved transcriptional regulatory mechanisms of host cell metabolism during viral infection [83].

In 1925, ILTV was first reported in the United States [84]. ILTV is highly contagious and can cause severe respiratory diseases in chickens, pheasants, and peacocks, resulting in significant economic losses for the poultry industry. Mortality rates vary depending on the virulence of the strain’. Common clinical signs of ILTV infection include paralysis, torticollis, rhinorrhea, conjunctivitis, wheezing, and abnormal breath sounds. The virus can persist in the host for extended periods, affecting egg production in laying hens [85].

Autopsies of infected birds typically reveal fibrinonecrotic exudates, syncytial cell formation, and eosinophilic intranuclear inclusions. Research indicates that older birds are primarily affected by the disease, and outbreaks are often associated with vaccine strains, possibly due to virulence reversal or transmission between birds [86]. Recombination between strains can lead to the emergence of virulent and transmissible strains [87].

**Figure 5:**
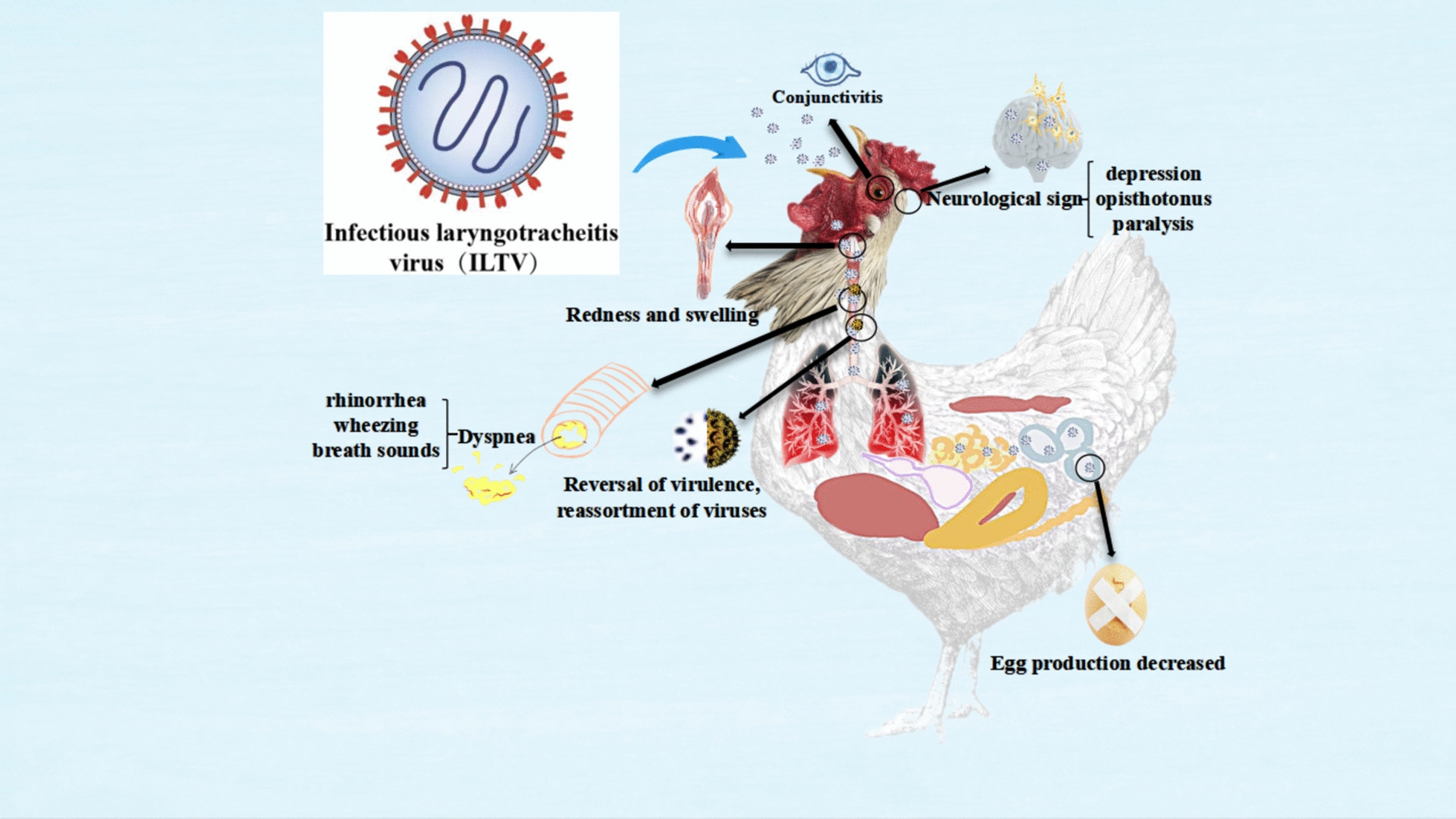
**The primary respiratory symptoms caused by ILTV.**

The prolonged shedding period, changes in virulence, and strain recombination pose significant challenges for preventing and controlling ILTV. Figure 5 shows the viral structure of ILTV and the clinical signs and pathological changes caused by ILTV in chickens. Recent reports from Switzerland indicate that 21 ILTV samples from natural outbreaks contained both vaccine-like and wild-type ILTV strains, with ongoing mutations observed in the region [88]. The various virus strains exhibit a high level of genetic diversity, which may affect their pathogenicity and transmissibility.

Furthermore, recent findings from Hubei Province in China reveal that the wild strain of ILTV is gradually evolving into the chicken embryo origin (CEO) vaccine strain [89]. Reports from Turkey between 2018 and 2022 documented that chickens exhibited severe respiratory symptoms in confirmed ILT cases [90]. Sequence analysis has shown that ILTV possesses significant genetic diversity, allowing it to continue evolving in natural environments and potentially leading to reduced vaccine effectiveness.

Due to this genetic variation, existing vaccines may not adequately protect all poultry, especially against virus strains with high mutation rates. A molecular flow survey of laying hens in Bangladesh conducted in 2021–2022 supported these findings [91]. Additionally, a meta-analysis of ILTV studies from China suggested that differences in ILT prevalence across regions can be linked to factors such as feeding practices, stocking density, and the use of live vaccines [92].

The incidence of ILT is partially attributed to the use of live vaccines, even in Taiwan, where they have largely replaced wild-type viruses as the primary cause of epidemic outbreaks. Immune escape due to vaccination poses a significant risk for preventing and controlling ILTV.

### Avian metapneumovirus

aMPV is classified within the family *Paramyxoviridae,* subfamily *Pneumovirinae*, and genus Pneumovirus. It is an unsegmented, single-stranded, negative-sense RNA virus, measuring approximately 13 kb in length and enclosed in a nucleoprotein shell with helical symmetry [93]. The structural composition of aMPV particles is generally spherical and similar among nonvirininae members.

This virus lacks non-structural proteins and has a gene sequence of 3'leaderN-PM-FM2-SH-GL-trailer-5', encoding the nuclear protein, phosphorylated protein, membrane protein, fusion protein, second membrane protein, and small hydrophobic protein, as well as accessory glycoproteins and RNA polymerase. The N protein serves as an RNA-binding protein and is part of the polymerase complex [94].

The F, G, and SH proteins are the envelope proteins of aMPV, with the F and G proteins being the primary antigenic structural proteins. The F protein is responsible for membrane fusion, host tropism, and immunogenicity enhancement, while the G protein acts as a mutable gene that influences viral replication and acquired immunity [95].

aMPV is categorised into four subtypes based on the G accessory protein gene: aMPV-A, aMPV-B, aMPV-C, and MPV-D. Subtypes A and B have a broad prevalence, with limited cross-reactivity between different subtypes. Subtype C shows a closer genetic relationship to human metapneumovirus, also classified under the metapneumovirus group. This distinction has led to the categorisation of metapneumovirus into two types: type I (comprising aMPV A, B, and D subtypes) and type II (comprising aMPV C and human metapneumovirus) [96].

Recent studies have shown that the F protein of aMPV can induce immune protection. However, aMPV/C suppresses the production of interferon beta by host cells through its F protein, which weakens the host’s innate antiviral immune response [97]. Additionally, infection with aMPV/C triggers the degradation of mitochondrial antiviral signalling through the ubiquitin–proteasome pathway [98]. Furthermore, autophagy has been found to impede the replication of aMPV/C, with the molecular mechanism of autophagy induction relying on the modulation of the endoplasmic reticulum stress-associated UPR pathway [99].

aMPV was first identified in South Africa in 1978 and was categorised as a pneumovirus in 1986. Various subtypes, particularly A and B, have been found in turkeys and chickens worldwide. Subtype C was discovered in the United States in 1997, followed by the identification of subtype D in France [96].

aMPV significantly contributes to acute respiratory diseases in poultry, adversely affecting egg production. In adult chickens, the infection typically results in mild symptoms such as depression and reduced appetite. In laying hens, it may lead to reproductive tract issues, which can diminish egg production and cause yolk-related conditions and organ degeneration. Severe coughing can result in fallopian tube abnormalities [100].

Affected birds may also exhibit symptoms such as swollen head syndrome, turkey tracheitis, and avian tracheitis. Swollen head syndrome can present as mild respiratory or neurological symptoms, including opisthotonus. However, not all cases are due to aMPV, as other infections, such as *E. coli* and infectious bursal disease, can cause similar clinical signs [101].

Turkey tracheitis typically presents with mild to moderate upper respiratory symptoms. The primary clinical signs of aMPV infection in turkeys or chickens typically involve mucoid discharge from the nasal cavity and eyes, as well as a yellow or white cheese-like discharge in the nose or trachea upon post-mortem examination [102]. These symptoms often result from aMPV targeting ciliated columnar epithelial cells in birds’ upper respiratory tract, leading to inflammation of the trachea and lungs.

This inflammation is characterised by damage to the epithelial structure, shedding of cells, shedding of the epidermal layer, infiltration of inflammatory cells, bleeding, lymphoid cell infiltration, and the proliferation of the tracheal epithelium and lamina propria [101]. Furthermore, slight dilation of the alveolar interstitium may occur due to mononuclear cell infiltration and oedema.

Histopathological examination of the lungs may reveal peribronchial lymphoplasmacytic infiltration, bronchial submucosa oedema and thickening, lymphoid cell proliferation, as well as the presence of shed epithelial cells, macrophages, and irregular debris in the bronchial lumen [101].

**Figure 6:**
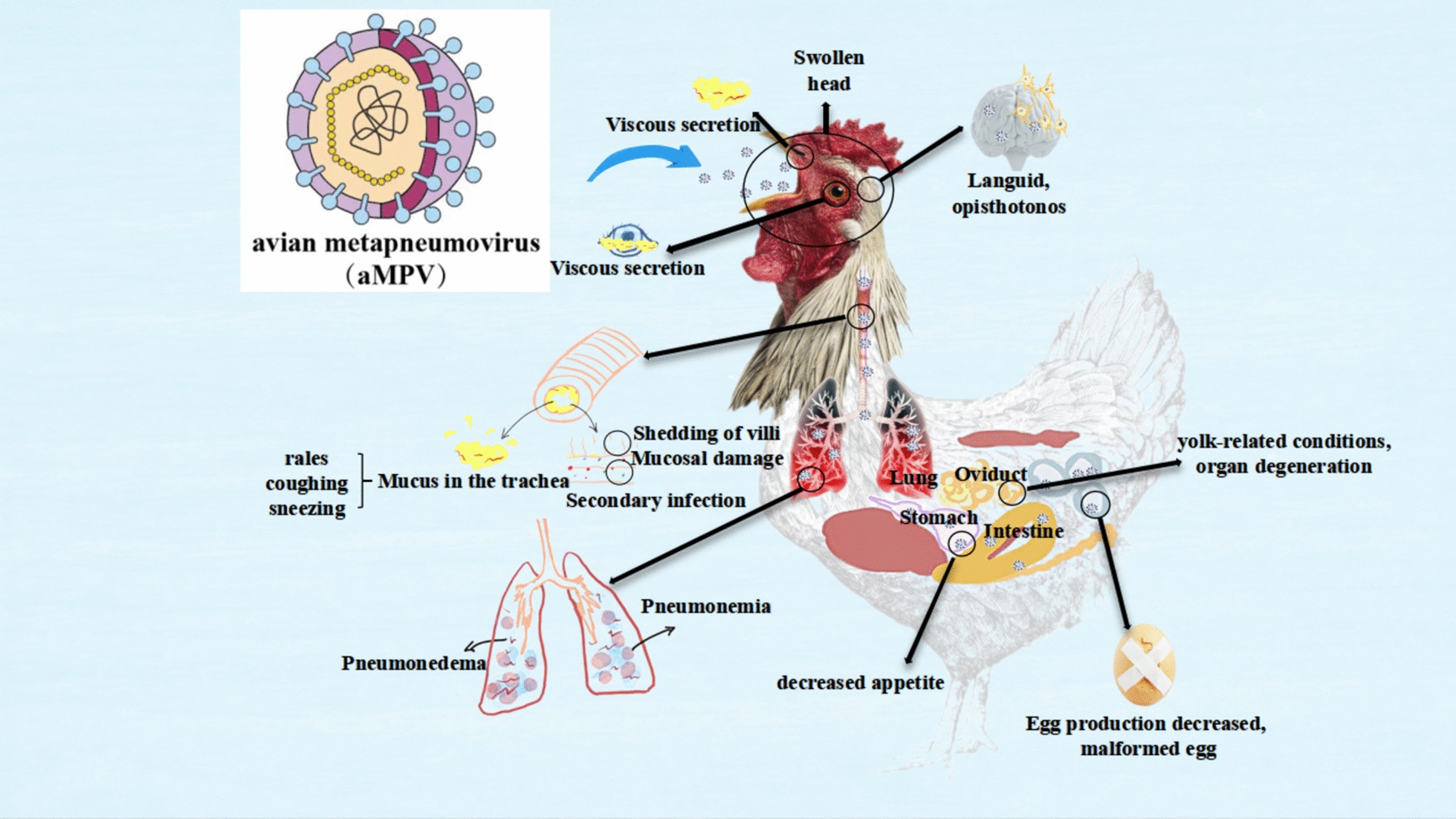
**The primary respiratory symptoms caused by aMPV.**

Figure 6 illustrates the structure of the aMPV and its effects on chickens suffering from clinical signs and pathological lesions caused by aMPV.

### Avian pathogenic Escherichia coli

APEC is a Gram-negative bacterium characterised by peritrichous flagellar movement and its specific virulence structures. The Ewing typing system classifies *E. coli* into various serotypes based on 180 O antigens, 60 H antigens, and 80 K antigens [103]. The O antigen serves as a crucial immune stimulant in poultry, making research on APEC particularly important. Common serotypes of APEC include O1, O2, O35, O78, O15, and O55 [104]. Although APEC is typically non-intestinal, it can inhabit the intestine and cause infections under specific environmental conditions. APEC can lead to secondary infections and direct diseases, with its pathogenicity arising from virulence factors acquired through horizontal gene transfer [105]. These factors, which can be found in bacterial chromosomes, plasmids, or virulence islands, include outer membrane proteins, adhesins, serum resistance proteins (Iss), anti-phagocytic factors, iron uptake systems, hemolysin, colicin, and lipopolysaccharide.

The respiratory tract is a common entry point for APEC, which interacts with the respiratory microbiota [106]. Lung epithelial cells are the primary target of APEC and trigger innate immune responses upon infection. Research shows that pulmonary infection with avian pathogenic *E. coli* leads to the simultaneous upregulation of cSP-A and cLL proteins, which are both involved in innate immunity within chickens. Furthermore, APEC infection can induce pathological changes in the chicken trachea, as evidenced by the altered mRNA expression of genes related to inflammation and proliferation signalling pathways, highlighting the regulatory mechanisms by which APEC affects the pathogenicity of the chicken’s tracheal epithelium [107].

The chicken immune system recognises various pathogen molecular patterns, including peptidoglycan, lipopolysaccharide, flagellin, and non-cylated CpGDNA, through specific Toll-Like Receptors (TLRs) such as TLR-2, TLR-4, TLR-5, and TLR-21. APEC triggers inflammatory responses and phagocytic activities in the lungs via the TLR receptors on epithelial cells and macrophages. Giant cells and eosinophils play crucial defensive roles in these responses. Experiments involving the tracheal inoculation of *E. coli* 506 have demonstrated an increase in the number of giant cells [108].

Heterophils, the predominant granulocytes in poultry, actively participate in immune responses against pathogens through the use of cytotoxic molecules and chemokines. In a model of APEC lung infection, heterophils serve as primary bacterial clearers but may also contribute to persistent inflammation, potentially causing tissue damage and influencing disease progression. While heterophils and giant cells are key players in the initial immune response, ongoing research on natural killer cells (NK cells) and their receptors suggests that they may also have significant roles during the early or late stages of infection [109–111].

Infection in chickens is characterised by acute and subacute sepsis. Acute sepsis, caused by yolk sac and respiratory infections, can lead to death, while pericarditis, peripheral hepatitis, and airsacculitis are common outcomes of subacute sepsis [112]. APEC is widely found in the intestinal tracts of animals worldwide, including chickens, where it exists in high bacterial concentrations within faeces. APEC can be classified into pathogenic and non-pathogenic strains, with the host’s physiological state influencing susceptibility to the bacteria. Research has shown that *E. coli* can invade the lungs through the damaged air sac interstitium, utilising various protective proteins, such as the K1 capsule, ISS, T2SS, and O78LPS, to evade the host’s complement system and thrive in the bloodstream [113].

**Figure 7:**
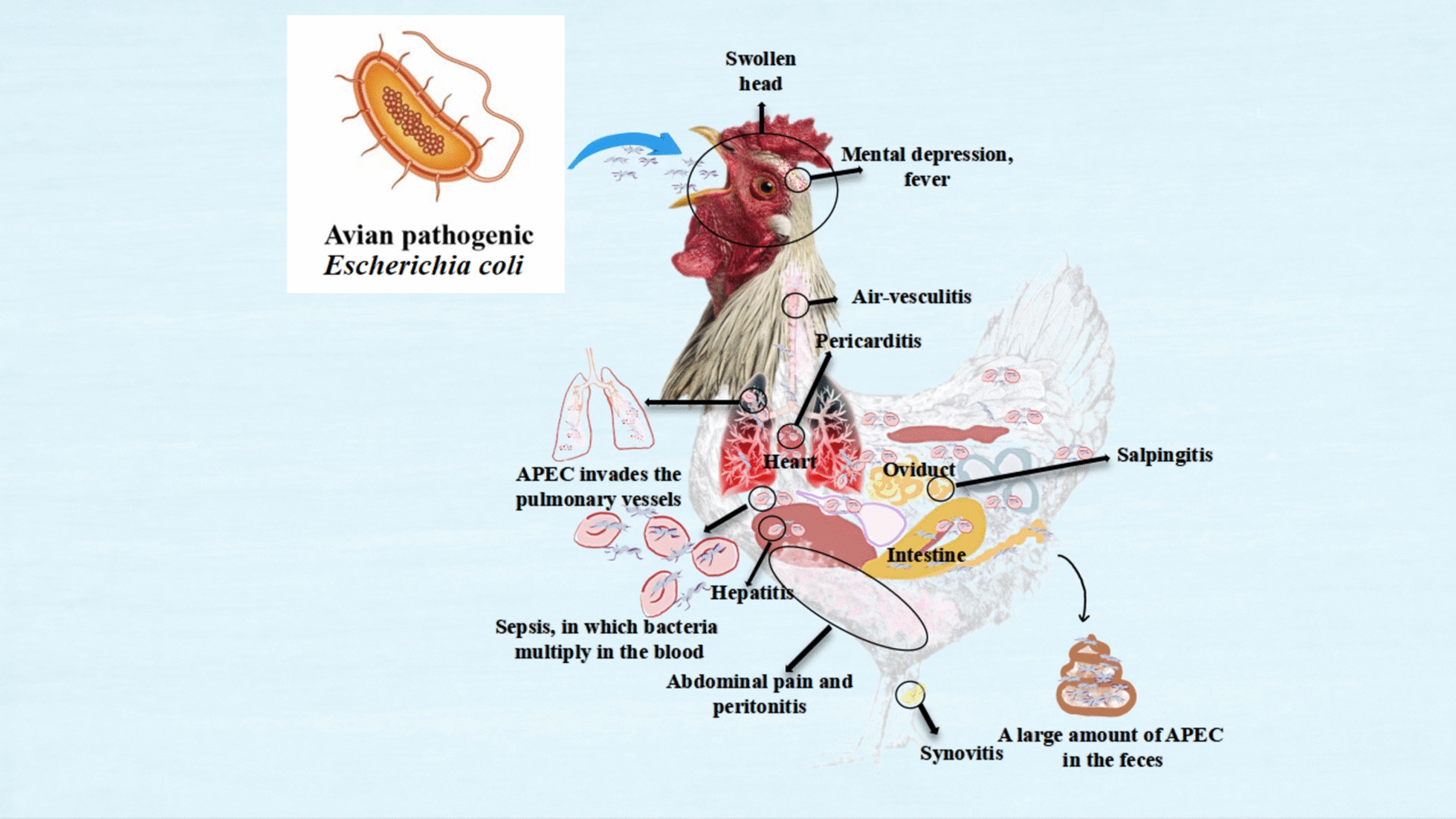
**The primary respiratory symptoms caused by APEC.**

Once the bacteria invade the tissue, an acute inflammatory response occurs, characterised by an increase in acute phase proteins, cytokine-1, IL-6, and tumour necrosis factor, indicating early pathological changes [114]. The clinical signs of APEC infection can vary and may include abdominal pain, inflammation, swollen head syndrome (common in broilers), peritonitis, salpingitis, synovitis, and sepsis. Swelling may also result from upper respiratory tract viral infections secondary to APEC [115]. Salpingitis may result from an APEC infection in the cloaca and left abdominal air sac in laying hens and broilers. Sepsis is particularly severe in broiler chickens, exhibiting symptoms such as depression, fever, and cardiac lesions, including pericarditis and synovitis, making it a significant cause of mortality in APEC-affected populations. Figure 7 illustrates the strain morphology of APEC and the clinical signs and pathological lesions it causes in chickens.

### *Avianbacterium paragallinarum*

*Avianbacterium paragallinarum* (Apg) is a Gram-negative, non-motile, small bacillus measuring 1–3 μm in length and 0.4–0.8 μm in width. It has densely stained poles and does not produce spores. The highly virulent strains of Apg possess a capsule, which can be easily lost during in vitro subculture.

Traditional typing methods for Apg serotypes include the glass plate agglutination test, which divides them into three main serotypes: A, B, and C, each exhibiting unique immunogenic properties. However, some isolates remain untyped [116]. Another method used is the Hemagglutination Inhibition test, which categorises Apg into three serogroups and seven serotypes [117].

Studies on Kume serotypes have indicated good homologous cross-protection among type A and C strains. However, immune responses vary significantly across serogroups A, B, and C, with limited cross-protection observed between types A and B [118]. The pathogenicity of Apg varies based on the isolate type, host status, and environmental factors. Some Kume serotype strains demonstrate higher pathogenicity, with NAD-independent isolates more likely to cause airsacculitis and NAD-dependent isolates have shown increased virulence in studies conducted in South Africa [119]. Thus, the pathogenicity of Apg is multifactorial and includes the influence of hemagglutinin antigens.

The hemagglutinin antigen, particularly the major outer membrane protein HMTp210, is essential for colonisation and pathogenesis of Apg. It facilitates the attachment of the bacteria to host cells [120, 121]. Additionally, the capsular polysaccharide of *I. paragallinarum* plays a significant role in bacterial colonisation and protection against the bactericidal activity of chicken serum. High-performance liquid chromatography analysis shows that the capsular polysaccharide primarily consists of d-xylose, d-glucose, and L-rhamnose [122]. Plasmids in these bacteria may carry virulence-related genes, such as the AvxA gene, which encodes the RTX toxin that demonstrated cytotoxicity towards the chicken macrophage cell line HD11 in a 2013 study. This gene is present in all serotypes of Apg [123]. These findings emphasise the importance of considering these key factors in the development of vaccines and strategies for disease prevention and control.

Chicken infectious rhinitis is an acute upper respiratory tract infection caused by Apg. Chickens may show symptoms such as depression, swelling of the eyelid, head, and face, coughing, and increased viscous secretion within 1 to 2 days after infection. Some chickens may also experience diarrhoea, decreased appetite and water intake, and, in severe cases, swollen eyes. Some of these symptoms may then result in weight loss [124].

Prolonged infections can lead to higher culling rates and reduced egg production. Co-infection with other pathogens, such as MG or infectious bronchitis, can worsen the condition and increase mortality rates. This disease can affect meat quality in broiler chickens and increase mortality.

The Apg infection primarily causes catarrhal inflammation in the nasal cavity and sinuses of chickens, resulting in mucous and purulent secretions. Severe cases may display yellow-white matter in the sinus cavity, laryngeal and tracheal lesions, and occasionally pneumonia. Common manifestations include subcutaneous oedema, yellow-white exudate in the eyelids, and mucosal changes in the upper respiratory tract. These changes, such as mucosal shedding, cell damage, and vascular issues, contribute significantly to the overall pathology of the disease [125].

**Figure 8:**
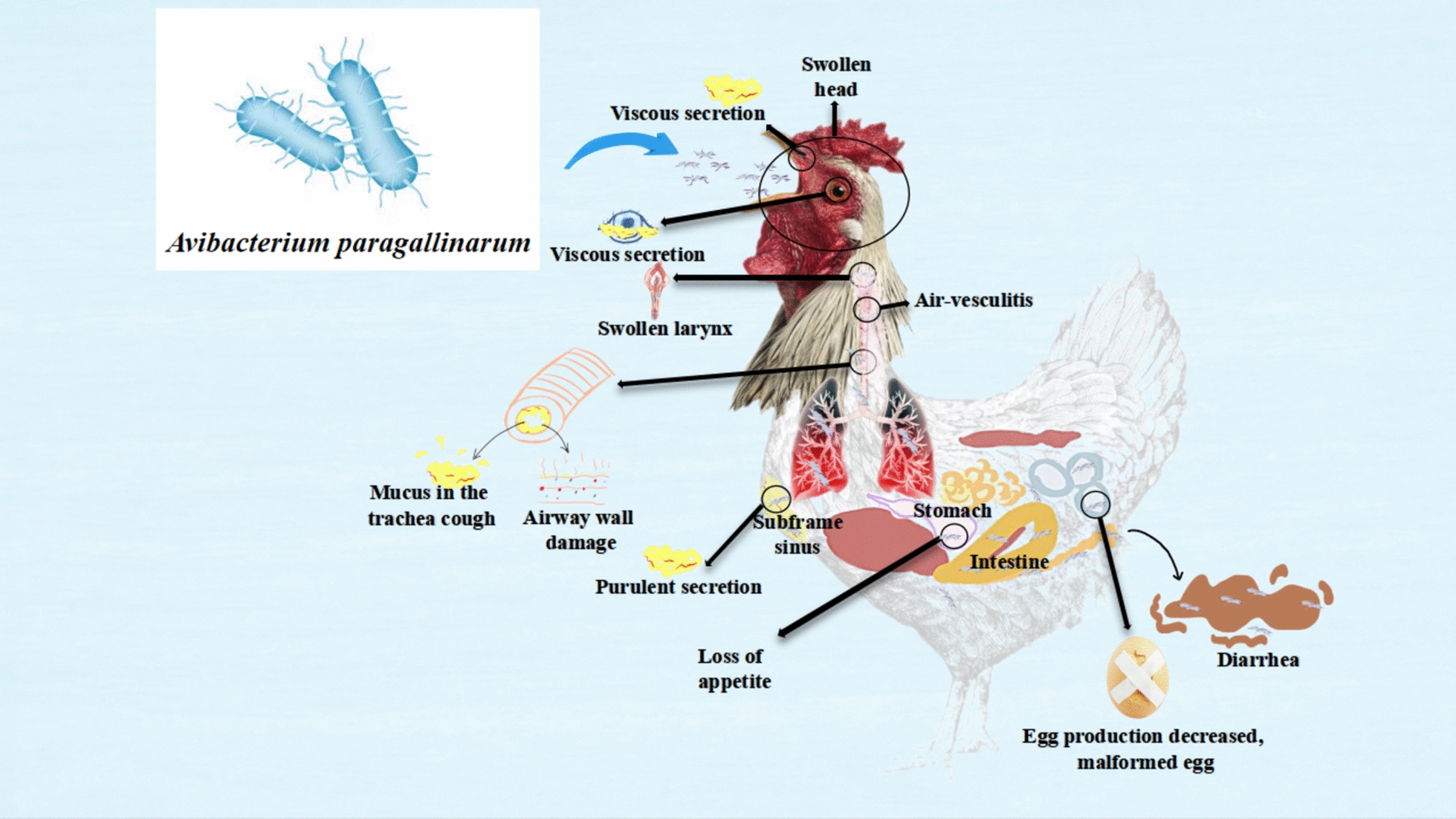
**The primary respiratory symptoms caused by Apg.**

Figure 8 illustrates the morphology of the Apg strain and the condition of the chicken after experiencing the clinical signs and pathological lesions associated with this infection.

### *Mycoplasma*

*Mycoplasm*a is a prokaryotic microorganism that belongs to the class Mollicutes. It is characterised by its lack of a cell wall and its small size, and it is capable of self-replication [126]. *Mycoplasma* can be found in various environments, including soil, plants, insects, poultry, mammals, and humans. It reproduces through binary fission or budding, forming characteristic “poached egg-shaped” colonies on solid culture media.

Several pathogenic strains of *Mycoplasma* affect poultry, including MG, *Mycoplasma meleagridis* (MM), *Mycoplasma synoviae* (MS), and *Mycoplasma iowae* (MI). Among these, MG is a significant pathogen that causes chronic respiratory diseases in poultry. It exhibits variations in pathogenicity and tropism among isolates from different regions [127].

The MG genome encodes multiple proteins essential for pathogenesis and immune response induction. These proteins encompass virulence factors, adhesion molecules, lipoproteins, heat shock proteins, and antigenically variable surface proteins [128].

The absence of a cell wall in MG emphasises the importance of lipid-associated membrane proteins (LAMPs) present on its surface during the early stages of host invasion and the initiation of infection. These proteins interact with adhesion proteins on host cells, activating various signalling pathways that lead to inflammatory responses, which are crucial for the pathogenesis of MG [129].

Toll-like receptors (TLRs) are a group of highly conserved pattern recognition receptors that play a crucial role in recognising specific molecules within LAMPs. They activate innate immune cells to initiate inflammatory responses, which are essential for combating MG invasion [130]. Research shows that MG LAMPs comprise hydrophobic proteins and lipoproteins that stimulate the NF-κB signalling pathway through TLR1, TLR2, and TLR6 receptors. This stimulation prompts monocytes and macrophages to release immature IL-1β, triggering an immune response [131].

The interaction between *Mycoplasma* and TLR2 receptors enhances the expression of adhesion proteins in epithelial cells, leading to Th2-type immune responses. Additionally, LAMPs from pathogenic microorganisms bind to TLRs, activating NF-κB and AP-1 transcription factors through various signal transduction pathways to regulate downstream cytokine production, including IL-1β [132].

The adhesion mechanism of MG mirrors that of *Mycoplasma pneumoniae* as it utilises fibronectin-binding proteins and heparin-binding proteins to facilitate adhesion. *Mycoplasma* also thwarts host cell apoptosis through distinct structural components, allowing it to avoid immune system clearance and further invade host cells. Furthermore, *Mycoplasma* can induce pathological changes in host organs by prompting apoptosis, which results in clinical symptoms [133].

The relationship between *Mycoplasma* infection and cell apoptosis is significant. Different *Mycoplasma* species induce apoptosis differently and can disrupt normal host cell functions through multiple pathways, ultimately leading to cell death. Although progress has been made in understanding MG pathogenesis, the mechanisms underlying the interaction between MG infection and respiratory flora require further investigation.

MG primarily infects chickens and turkeys but can also affect various other poultry species, including pigeons, ducks, quails, and sparrows [134]. Clinical signs of respiratory disease caused by MG include dishevelled feathers, loss of appetite, lethargy, runny nose, coughing, tracheal rales, mouth breathing, eyelid swelling, and excessive tearing [135]. In chicks, MG infection often leads to growth retardation, increased mortality, and compromised immune function.

Laying hens infected with MG experience reduced production performance, including lower egg production and decreased egg quality. Broiler chickens may suffer weight loss of 20% to 40%, deterioration in meat quality, reduced feed utilisation efficiency by about 20%, and increased medication costs [136]. Although the mortality rate due to MG alone is typically low, it can predispose birds to secondary infections from pathogens such as Newcastle disease virus and *E. coli*, which increases risks within the poultry industry.

MG spreads through horizontal and vertical transmission within chicken flocks, persisting throughout the entire breeding period and proving challenging to eradicate completely [137]. Poultry of all ages can be infected with MG infection, with cases occurring throughout the year, particularly peaking during the transitions between winter and spring, as well as autumn and winter. Environmental factors significantly influence the prevalence of MG. Overcrowding on chicken farms can weaken immune defences and facilitate the spread of MG. Additionally, excessive temperatures, humidity, and harmful gases like ammonia and hydrogen sulfide in chicken houses can damage respiratory mucosal barriers, leading to a higher risk of MG infection.

**Figure 9:**
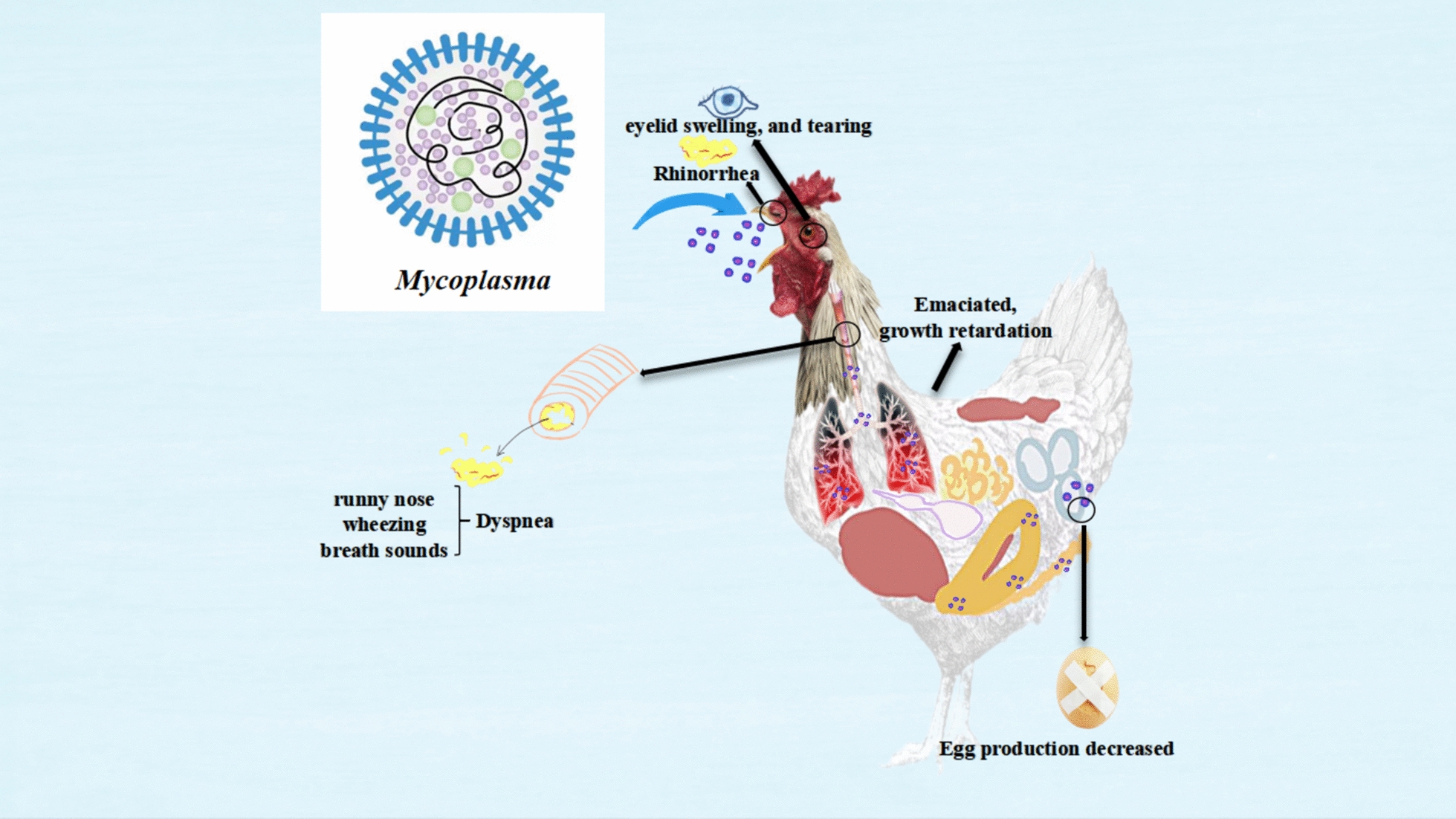
**The primary respiratory symptoms caused by MG.**

Figure 9 illustrates the pathogenic structure of MG, along with the clinical signs and pathological changes it causes in chickens.

### *Chlamydia*

*Chlamydia* refers to a class of intracellular parasitic prokaryotes that possess both DNA and RNA and reproduce through binary fission. Molecular genetic analysis has revealed that the 16SrRNA genes show homology that classifies them within the *Chlamydia* phylum, which includes 11 identified *Chlamydia* species [138]. Various poultry species have been found to harbour *Chlamydia*, with infections being asymptomatic but still capable of causing respiratory, intestinal, and ocular diseases under certain conditions.

Avian Chlamydiosis (AC) is primarily caused by *Chlamydia psittaci* (Cps), which has nine classic genotypes (A ~ F, WC, E/B, and M56), each with distinct host preferences and virulence levels [139]. The most common isolates in chickens are from genotypes B, C, and D. The ompA gene encodes the major outer membrane protein (MOMP) of approximately 40 kDa. MOMP is rich in cysteine and constitutes about 60% of the outer membrane. It contains both genus- and species-specific antigenic determinants in conserved regions and serotype-specific epitopes in its four variable regions. This makes MOMP a frequent target for genotyping Cps [140].

Recent studies have introduced a new typing method called multi-locus sequence typing, which utilises differential fragments of seven core genes (enoA, fumC, gatA, gidA, hemN, hflX, and oppA) of Cps [141, 142].

*Chlamydia* has developed mechanisms to evade hostile conditions by transforming into intermediates characterised by an enlarged primordial morphology, which enhances its long-term survival within host cells. As an intracellular microorganism, *Chlamydia* also influences the cell growth cycle, metabolism, and antigen presentation in host cells. It undergoes two bidirectional development cycles with distinct morphological structures [141, 142] that produce both infectious elementary bodies (EB) and non-infectious reticulate bodies (RB).

The EB is a protein shell composed of various proteins cross-linked by cysteine disulfide bonds, forming the *Chlamydia* outer membrane complex (COMC). This complex contains major outer membrane proteins, cysteine-rich proteins, and polytypic membrane proteins [143]. EBs have low metabolic activity, condense their DNA into nucleoids, and enter host cells via the type III secretion system. Once inside, EBs transform into RBs to evade degradation by phagosome-lysosomes. RBs replicate through binary fission, creating inclusion bodies (IB) within the host cells. Upon reaching a certain threshold, RBs convert back into EBs and are released to initiate new round of infection.

Moreover, *Chlamydia* shares common metabolic pathways with other bacteria. The Cps protein has been shown to regulate oxidative stress in human bronchial epithelial (HBE) cells through the miR-184 and Wnt/β-catenin signalling pathways [144]. In Cps-infected HeLa cells, there was a significant increase in the mRNA levels of JAK1 and STAT3, as well as elevated levels of total and phosphorylated JAK1 and STAT3 proteins [145]. The plasmid protein CPSIT_p7 of Cps can activate RAW264.7 cells, leading to the expression of primary regulatory factors such as LC3 and Beclin1. This activation triggers phosphorylation of ERK, JNK, p38 MAPK, and AKT, leading to sequential activation of the TLR4/Mal/MyD88/NF-κB signalling axis, which culminates in the production of inflammatory cytokines [146].

Additionally, the N terminus (PmpD-N) of the polymorphic membrane protein D (PmpD) found in Cps can inhibit macrophage function by activating the Th2 immune response and the TLR2/MyD88/NF-κB signalling pathway [147]. The hypothetical inclusion membrane protein CPSIT_0842 of Cps can increase the expression of IL-6 and IL-8 in THP-1 cells through the activation of TLR-2/TLR4-mediated MAPK and NF-κB signalling pathways, ultimately leading to macrophage apoptosis induced by MAPK/ERK autophagy [148]. Furthermore, exposure to the secreted protein SINC of Cps can induce autophagy in RAW264.7 cells by activating the MAPK/ERK signalling pathway [149].

The source of infection, transmission routes, and susceptible animals mainly influence *Chlamydia* epidemics. Various factors, such as the types of animals, vectors, strain variations, environmental conditions, and management practices, contribute to the complexity and variability of the epidemic.

Cps is a widely distributed species that can infect over 450 species of poultry [150]. While most infections are asymptomatic, clinical manifestations can vary due to differences in pathogenicity among strains [151]. Additionally, clinical symptoms may be affected by factors such as poultry breed, age, nutritional status, immune response, and the environment.

Cps is primarily transmitted through faeces and respiratory droplets, often leading to latent infections that are difficult to diagnose. Co-infection with other pathogens can result in severe clinical symptoms and high mortality rates. Cps can parasitise poultry of various ages, causing issues such as hydrosalpinx in laying hens, decreased egg production, slow growth in broiler chickens, and increased chick mortality [152].

**Figure 10:**
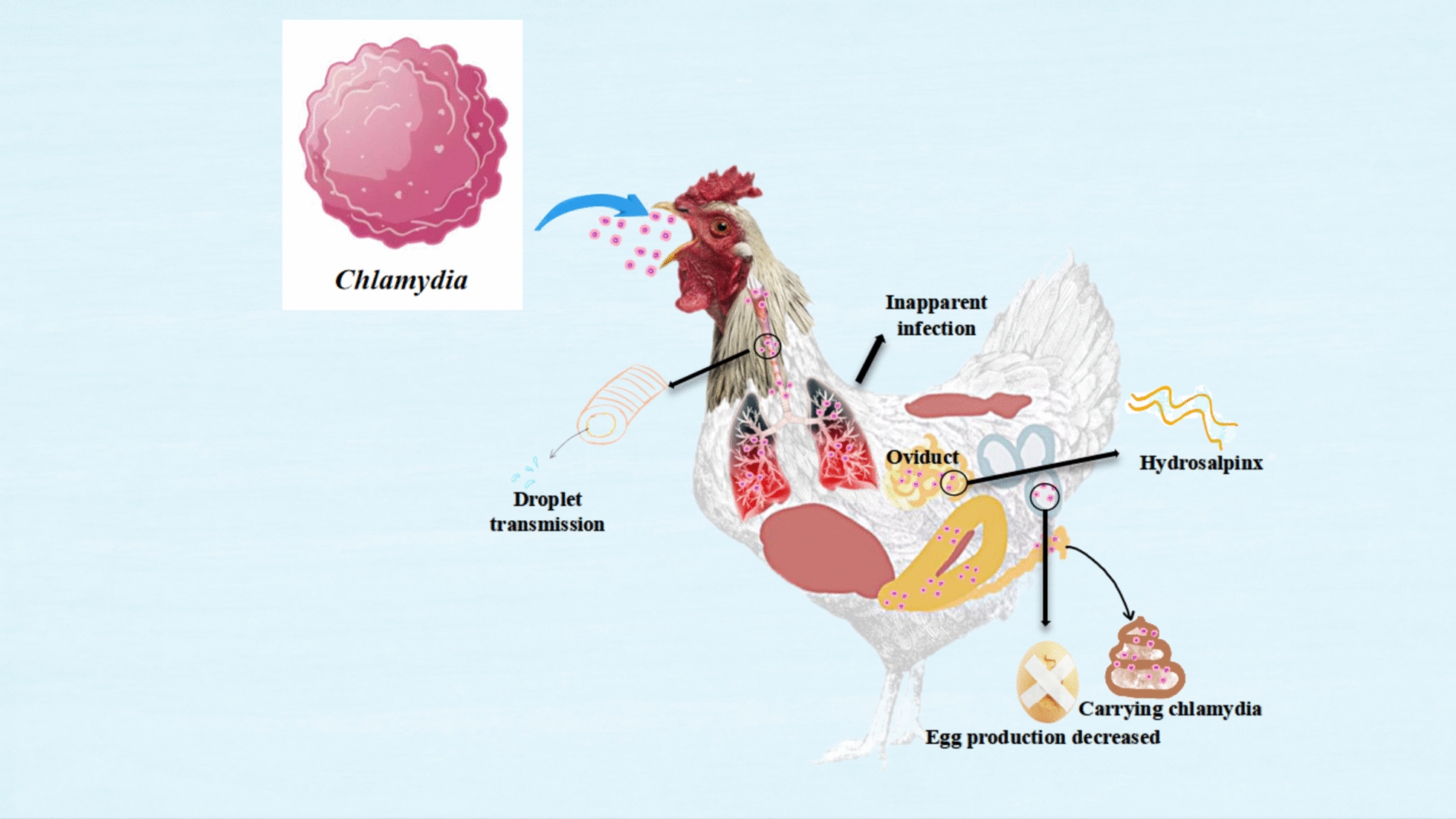
**The primary respiratory symptoms caused by Cps.**

Infected chickens can shed *Chlamydia* for extended periods, making them significant sources of infection. Figure 10 illustrates the pathogenic structure of Cps and the clinical signs and pathological lesions it causes in chickens.

## Analysis of non-infectious factors

### Environmental factors

Fine particulate matter (PM) in poultry houses primarily comes from biological sources, such as poultry feathers, mineral crystals found in urine, and waste materials [153]. PM concentration is influenced by several factors, including poultry species, housing practices, stocking density, environmental control systems, humidity, season, and sampling time.

PM can irritate the respiratory tract, weaken the immune system of poultry, and lead to respiratory diseases. Research shows that PM of different sizes exhibits varying deposition levels in the respiratory tract of poultry. Specifically, PM with smaller diameters (less than 20 microns and 1 micron) tends to deposit more in the lungs of poultry, increasing the risk of respiratory damage [154]. Some studies suggest that poultry may display enhanced immune responses in high-concentration PM environments, indicating that PM could potentially trigger the immune system [155].

Prolonged exposure to PM could have lasting effects on lung health. Key components of PM, such as ammonia and hydrogen sulfide, can be harmful. Ammonia is a noxious gas that can reach hazardous levels in enclosed poultry houses. It damages mucosal barriers and can lead to immune dysregulation and inflammatory responses. Poultry chronically exposed to ammonia show elevated inflammatory markers, increased apoptosis, and various immune disorders. Additionally, ammonia can alter the structure of the respiratory microbial community, leading to further inflammation [156].

Another common harmful gas, hydrogen sulfide, is frequently found in biogas, sewage outlets, and similar environments. Chickens exposed to high levels of hydrogen sulfide may experience respiratory issues such as breathing difficulties and lung damage. Prolonged exposure can also compromise their immune systems, making them more vulnerable to respiratory diseases and potentially life-threatening conditions.

Inadequate ventilation is a common issue in chicken houses, leading to elevated levels of harmful gases and particulate matter. A lack of fresh air can reduce the oxygen supply, putting a strain on the respiratory system and adversely affecting the health and performance of chickens. The accumulation of gases such as ammonia and hydrogen sulfide, along with fine particles, can irritate the chickens’ respiratory mucosa, leading to inflammation and various diseases [157]. Furthermore, the deposition of particulate matter can impair lung function and hinder oxygen exchange. Sufficient oxygen is crucial for the normal function and growth of chickens, particularly in high-density conditions. Insufficient oxygen can overwhelm the respiratory system, decrease efficiency, weaken the immune response, and increase susceptibility to respiratory illnesses [158].

Maintaining an appropriate microclimate within the poultry house is essential for chickens’ health. Improper temperature and humidity levels can negatively affect their respiratory system, making them more susceptible to diseases [159].

High temperatures can hinder chickens’ ability to regulate their body temperature, causing an increased respiratory rate as they attempt to cool down. This strain on their respiratory system can, in hot conditions, irritate the mucosal lining of the respiratory tract, making it more vulnerable to bacterial and viral infections, thereby increasing the likelihood of respiratory illnesses [160].

On the other hand, low temperatures can slow down a chicken’s metabolism, leading to a reduced respiratory rate as they conserve heat. However, extremely low temperatures can also irritate the respiratory mucosa and airways due to the cold air, increasing the risk of respiratory infections and inflammation, which ultimately harms respiratory health [161].

Additionally, humidity levels that are either too high or too low can impact chickens’ respiratory health. Excessive humidity can facilitate the spread of bacteria and viruses in the air. In contrast, low humidity can lead to dryness and damage to the respiratory mucosa, further increasing the risk of respiratory diseases in chickens.

### Improper management

Overcrowded housing is a common issue that can lead to poor air quality, increased rates of disease transmission, and elevated stress levels. In densely populated breeding environments, chickens often do not have enough space or access to fresh air, making them more susceptible to respiratory and infectious diseases that can harm their health and productivity [162]. Furthermore, overcrowded conditions can result in higher concentrations of harmful gases like ammonia and hydrogen sulfide, as well as increased levels of bacteria in the air, significantly decreasing air quality. Prolonged exposure to such environments can damage the chickens’ respiratory mucosa and cause fatigue, further increasing their vulnerability to respiratory issues.

Additionally, high stocking densities can create competition and stress among chickens, negatively affecting their physiological and immune functions [162]. This heightened competition can lead to stress-induced immunosuppression, reducing their ability to fight off diseases. As a result, chickens in high-stress environments face a greater risk of respiratory illnesses, and diseases tend to spread more quickly in densely stocked settings.

Inadequate hygiene in cages, feeding equipment, and drinking water systems can lead to the buildup of pathogens, which poses a respiratory health risk to chickens. Unclean equipment can become a breeding ground for bacteria, fungi, and viruses, increasing the likelihood of exposure to respiratory pathogens that can negatively affect the birds’ respiratory health. Cages and feeding utensils in unsanitary conditions may contain organic matter, such as bird droppings and feed residues, which fosters the growth of harmful microorganisms [163]. These pathogens can proliferate on equipment surfaces, making chickens more susceptible to infections. Additionally, poorly maintained drinking water systems can contaminate water sources, leading to polluted drinking water. Bacteria and contaminants in the drinking water can enter the birds’ bodies when they consume the water, causing respiratory and digestive tract illnesses.

## RS prevention and control

### Detection techniques of RS

When dealing with sick chickens, it is essential first to observe clinical symptoms such as dyspnoea, wheezing, runny nose, and eye secretions. In sick chickens, it is vital first to observe clinical symptoms such as dyspnoea, wheezing, runny nose, and eye secretions. Conducting necropsies on deceased chickens can provide valuable information by identifying pathological changes in the respiratory tract. A microscopic examination of tissue samples from the respiratory tract can reveal signs of inflammation and other pathologies [164].

Because various diseases may involve mixed or secondary infections, different pathogens can exhibit similar symptoms or may exist in subclinical forms. Therefore, utilising diagnostic tools that are sensitive, specific, accurate, and time-efficient is essential for effective disease identification.

Effective disease control measures are critical. Numerous methods have been developed and documented for identifying avian respiratory pathogens, including histopathology, virus isolation from embryonated eggs and cell culture, immunofluorescence (IF), immunoperoxidase (IP) assays, and serology [165–168].

Molecular diagnostic tools are widely being used as alternatives to traditional implantation methods due to their high specificity and sensitivity [169]. These tools are particularly valuable for confirming and quantifying viral loads in biological samples [18]. Diagnostic laboratories are developing and implementing various methods to enhance detection specificity.

While viral amplification and blood agglutination inhibition remain the gold standard for diagnosing viral respiratory diseases, they can be time-consuming, taking 7 to 14 days. ELISA kits can also improve detection specificity.

Identifying bacterial diseases often involves the use of specialised culture media. Studies have shown that molecular diagnostic assays, such as rt-PCR, offer higher sensitivity and faster results than traditional methods, positioning them as the new gold standard.

Researchers have developed a luminol suspension microarray method that employs multiplex PCR on a Flow Cytometer 200 analyser to rapidly detect clinically significant avian respiratory viruses. This method utilises single-channel or multiple-channel duplex laser scanning to swiftly identify four avian respiratory viruses (AIV, NDV, IVV, and ILTV) in single or mixed infections [170].

Based on the gene expression analyser, various studies have explored different molecular techniques to detect avian respiratory pathogens. One method, which involved multiplex PCR analysis based on Gexp analysis, achieved a 100% success rate in detecting nine pathogens [171]. Another innovative approach employed quantitative PCR with nanofluidic technology to identify 15 respiratory pathogens, including viruses, bacteria, and fungi associated with poultry respiratory infections. This method enabled simultaneous screening of 96 samples using 96 pathogen-specific PCRs in real-time quantitative PCR [25]. Additionally, multiple genotype detection technologies have been developed for single pathogens.

### Vaccine prevention and control of RS

In conjunction with research efforts to develop effective vaccines and preventive strategies, new approaches should be explored to enhance favourable host innate responses. This is important for inducing rapid innate antiviral responses to viral infections. Vaccination is a common strategy to control RS, but there is currently no universal vaccine available [172]. In addition to targeting viruses, a variety of vaccines are also being developed to prevent diseases caused by bacterial pathogens in poultry [173–176].

Vaccines for RSV include several types, such as inactivated, live attenuated, and recombinant vaccines. Various administration methods are used, including injections, eye drops, and sprays [4].

Inactivated vaccines are developed by deactivating pathogens, preventing them from causing disease when administered. However, these vaccines often elicit a limited immune response and have specific protective limitations. For example, the inactivated avian influenza vaccine for poultry requires two to three booster shots for laying hens and breeders. The optimal timing for administering various vaccines can also vary, primarily due to potential interactions between different vaccines. For instance, H9N2 can induce immunosuppression in poultry flocks, which may compromise the effectiveness of vaccinations against other pathogens [177, 178].

Live-attenuated vaccines are created by weakening the pathogens’ virulence to prevent disease while provoking an immune response. For instance, live vaccines with mutations in the crp gene targeting O78 in APEC and improved ILTV attenuated live vaccines targeting CEO and TCO have been developed [179]. These vaccines stimulate an immune response through tracheal infection without causing illness. Although the risk is minimal, there is a theoretical possibility that the pathogens in live attenuated vaccines could revert to their original virulence, especially in individuals with weakened immune systems. This could potentially lead to infection in unvaccinated flocks [180].

Stressful conditions, such as egg laying, transportation, and vaccination, can reactivate latent viruses in poultry, facilitating the spread of the disease. Additionally, spontaneous natural recombination between attenuated vaccines could give rise to new virulent strains [181]. Research on the effects of co-infection with IBV and ILT has predominantly focused on live IBV and ILT vaccines [182, 183].

Recombinant vaccines have distinct advantages, including their non-spreading nature, the inability to revert to virulent forms, and the absence of an incubation period. For example, these vaccines have been developed by expressing ILTV surface glycoproteins in vectors such as HVT, NDV, and fowlpox virus. These recombinant vaccines serve as effective alternative control strategies, providing over 90% protection [184].

While various vaccine candidates, including whole-organism and subunit vaccines, have been experimentally evaluated, there is still limited research on the protective efficacy of these commercial vaccines against colibacillosis [112]. Despite some promising findings, further investigation is essential before these vaccines can be commercially adopted in the poultry industry [18].

### Combination medication regimen

When implementing a combination drug strategy, it is crucial to consider potential interactions between different medications. It is vital to avoid combinations that may unintentionally inhibit or enhance the effects of other drugs unless such interactions are intended for therapeutic purposes. The selection and dosage of medications must comply with current legislation and regulations while prioritising the health and welfare of the birds.

Currently, management of chicken respiratory syndrome largely depends on the widespread use of antibiotics, often administered without a clear understanding of the specific pathogens involved [185, 186]. The prophylactic use of antibiotics and growth-promoting agents in feed has been a traditional preventive measure to address persistent issues. However, concerns have been raised regarding the increasing prevalence of antibiotic-resistant bacteria in the environment and antibiotic residues in food.

In treating chicken respiratory syndrome, especially in cases influenced by multiple factors such as viruses, bacteria, and environmental conditions, a combined drug strategy (multi-drug treatment) is typically considered more effective. This method aims to combat the disease through various mechanisms of action, alleviating symptoms while minimising the development of drug resistance.

Although fewer specific antiviral drugs are available for avian viruses, some may still be considered in certain situations, particularly for specific viral infections. Examples of such antiviral drugs include ribavirin, oseltamivir, and amantadine [187]. However, it is essential to note that some drugs, particularly ribavirin, are banned globally from use in animal husbandry.

Based on bacterial culture and sensitivity testing results, appropriate antibiotics can be selected for bacterial respiratory infections. Combinations of effective antibiotics against different bacterial classes, such as Gram-positive and Gram-negative bacteria, can cover a broader range of potential pathogens. Antibiotics like florfenicol, apramycin, and danofloxacin are approved for veterinary use against *E. coli* in chickens [188]. Among these, florfenicol is notable for its broad antibacterial spectrum and has received approvals from the U.S. Food and Drug Administration and many European Union members and China for treating respiratory diseases in poultry. Danofloxacin is approved in Asia, North America, and Latin America, primarily for bacterial and *Mycoplasma* diseases in poultry. It is crucial to consider the issue of bacterial resistance development when using these antibiotics.

At a commercial level, the poultry industry is actively seeking new technologies to combat infections in birds [112].

Using immune enhancers or immune modulators in chickens can significantly improve their immune system function, help combat pathogens, reduce inflammatory responses, and accelerate recovery. Vitamins A, D, E, and C each play unique roles in modulating both cell-mediated and antibody-mediated responses and offer immunomodulatory and anti-inflammatory effects. These vitamins aid the immune system in fighting microbial pathogens and decreasing stress-related risks while enhancing the response to vaccines. Additionally, selenium supplementation can lower oxidative stress in broilers and maintain meat quality [189]. Furthermore, beta-glucan, when included as a dietary supplement for poultry, has a notable positive effect on immune activity and intestinal morphology, serving as a biological modulator to enhance immune system capabilities [190].

### Demand for antibiotic alternatives

The food animal industry urgently needs to find alternatives to antibiotics due to increasing public health concerns about antibiotic resistance and restrictions on the prophylactic use of antibiotics [112]. As a result, many researchers are exploring various natural and synthetic alternatives to combat bacterial infections while evaluating their cost-effectiveness. Leading alternatives include probiotics, natural plants, and phages [191]. However, it is widely recognised that none of these options are as effective as antibiotic growth promoters in practical applications.

Currently, various immunomodulatory strategies are being investigated as alternatives to antibiotics in food animal production since these approaches do not lead to drug resistance. The innate immune system serves as a primary defence mechanism against various respiratory pathogens. In chickens, airway epithelial cells play a critical role in respiratory tract immunity by recognising and combating various pathogens, including bacteria and *Mycoplasma*. These cells help regulate the activity of surrounding immune cells, initiating systemic immune responses that are essential for defending against infections. Therefore, activating innate immune mechanisms represents an effective strategy for enhancing host resistance to respiratory diseases [192].

Herbal medicines represent a category of natural remedies that aim to minimise the use of preservatives, excipients, and other additives, thereby reducing potential side effects. Chinese herbal medicine employs Toll-like receptors (TLRs) to identify pathogen-related molecular patterns, such as cell wall components like LPS, peptidoglycan, bacterial DNA, and double-stranded viral ribonucleic acid. This recognition triggers innate immune responses to help prevent or combat pathogenic infections [193].

Studies have indicated that Astragalus Maxing Shigan Decoction, which comprises five herbs (almond, gypsum, ephedra, astragalus, and liquorice), provides antioxidant defence against ILT [18]. In birds treated with Yinhuang Erchen Mixture 72 h post-infection, higher concentrations of this mixture resulted in lower ILTV levels in tissues and improved mucosal immunity [194].

Rhein has shown promising in vitro antiviral activity against the Newcastle disease virus (La Sota strain IV) [195], while flavonoids from Sophora flavescens roots act as potent antiviral agents against the same virus [196]. Additionally, plant-derived indole and β-carboline alkaloids demonstrate significant antiviral activity against avian influenza viruses [197]. Furthermore, Spirulina exhibits bioactive and immunostimulatory properties, suggesting its potential as a dietary supplement for poultry to enhance growth, intestinal health, and disease resistance [198].

### Other strategies

Preventing various infections that can compromise the health of poultry is a crucial aspect of managing a poultry operation. To achieve this, poultry producers, veterinarians, and other relevant personnel have collaboratively established stringent biosecurity measures. These measures aim to prevent infections at every stage of the production process [112].

Key biosecurity strategies include situating chicken houses away from main roads, residential areas, and waste disposal points. It is essential to emphasise the importance of daily cleaning and disinfection of the chicken house, especially during empty periods. When empty, this cleaning process involves thorough cleaning, flushing, and disinfection of both the interior and exterior of the chicken house [199].

Since pests such as mosquitoes, flies, and mice are significant vectors for disease transmission, farms must regularly clean up faeces and ensure proper disposal of dead chickens. Effective measures must also be taken to control these pests and prevent them from spreading disease.

Monitoring the prevalence of Salmonella Enteritidis in chicken flocks can indicate the effectiveness of biosecurity measures. To prevent the entry of external pathogens, outsiders must be restricted from entering the production area. Farm employees should change into designated work clothes and shoes before entering the chicken house to avoid transferring contaminants between different housing areas.

Maintaining strict isolation measures, traffic controls, and thorough cleaning and disinfection can effectively reduce the intrusion of harmful bacteria and viruses [199]. In addition to these management strategies, researchers worldwide are exploring alternative methods to address bacterial infections in poultry.

While immunomodulators will be discussed in other sections, it is crucial to highlight that comprehensive control strategies—including timely disease diagnosis, the implementation of strict biosecurity protocols, thorough cleaning and disinfection, the use of geographic information system (GIS) technology, vaccinations and facilitating communication between poultry farmers and regulatory agencies—are all vital for preventing and controlling poultry diseases [200].

## Conclusions

Respiratory infections significantly impact poultry industry performance. This review discusses the negative impacts of viral and bacterial infections, as well as various rearing factors, on poultry respiratory systems. Recent reports from multiple countries have emphasised the damaging effects of synergistic interactions between different respiratory disease pathogens in poultry. The complexity of infection sources and secondary infections can make it challenging to monitor symptoms accurately.

Some detection technologies can swiftly identify various respiratory pathogens, and efforts are ongoing to optimise and improve them. Vaccines have been developed for several respiratory pathogens; however, challenges persist due to certain limitations of these vaccines. Poor vaccination practices, such as inadequate administration and the use of low-quality vaccines, can increase the risk of future outbreaks.

To combat respiratory infections on poultry farms, regular molecular surveillance of prevalent respiratory pathogens, stringent hygiene practices, effective management strategies, balanced nutrition, biosecurity measures, and appropriate vaccination protocols must be implemented. Proposed combination drug treatments for respiratory infections rely on molecular monitoring and vaccination histories to facilitate targeted interventions.

When utilising chemical drugs, it is crucial to consider issues relating to veterinary drug residues and drug resistance. Chinese herbal medicines are recommended as an alternative to antibiotics, as their active compounds can inhibit key processes involved in the replication and spread of respiratory pathogens, thereby reducing oxidative stress and inflammatory responses.

## Data Availability

This is a review article that synthesizes existing knowledge from previously published studies. All data cited in this work are available in the referenced original research articles, which are publicly accessible through the platforms.
